# Voices for food: methodologies for implementing a multi-state community-based intervention in rural, high poverty communities

**DOI:** 10.1186/s12889-018-5957-9

**Published:** 2018-08-23

**Authors:** Suzanne Stluka, Lindsay Moore, Heather A. Eicher-Miller, Lisa Franzen-Castle, Becky Henne, Donna Mehrle, Daniel Remley, Lacey McCormack

**Affiliations:** 1 Food and Families Program Director, South Dakota State University Extension, SWG 435, Box 2275A, Brookings, SD 57007 USA; 2 Moore Healthy Living, LLC, 4512 Southlea Dr, Winterville, NC 28590 USA; 3 Purdue University, 700 W. State Street, West Lafayette, IN 47907 USA; 4 University of Nebraska-Lincoln, 110 Ruth Leverton Hall, Lincoln, NE 68583 USA; 5 Michigan State University Extension, 551 Courthouse Dr. Suite 1, Charlotte, MI 48813 USA; 6 University of Missouri Extension, 1205 University Avenue, Suite 300, Columbia, MO 65211 USA; 7 Ohio State University Extension, 1864 Shyville Road, Piketon, OH 45661 USA; 8 South Dakota State University, Box 2203 SWG 445, Brookings, SD 57007 USA

**Keywords:** Food security, Food access, Nutrition, Community development, Community coaching

## Abstract

**Background:**

Rural communities experience unique barriers to food access when compared to urban areas and food security is a public health issue in rural, high poverty communities. A multi-leveled socio-ecological intervention to develop food policy councils (FPCs), and improve food security in rural communities was created. Methods to carry out such an intervention were developed and are described.

**Methods:**

A longitudinal, matched treatment and comparison study was conducted in 24 rural, high poverty counties in South Dakota, Indiana, Missouri, Michigan, Nebraska and Ohio. Counties were assigned to a treatment (*n* = 12) or comparison (n = 12) group. Intervention activities focus on three key components that impact food security: 1) community coaching by Extension Educators/field staff, 2) FPC development, and 3) development of a *MyChoice* food pantry. Community coaching was only provided to intervention counties. Evaluation components focus on three levels of the intervention: 1) Community (FPCs), 2) Food Pantry Organization, and 3) Pantry Client & Families. Participants in this study were community stakeholders, food pantry directors, staff/volunteers and food pantry clients. Pantry food access/availability including pantry food quality and quantity, household food security and pantry client dietary intake are dependent variables.

**Discussion:**

The results of this study will provide a framework for utilizing a multi-leveled socio-ecological intervention with the purpose of improving food security in rural, high poverty communities. Additionally, the results of this study will yield evidence-based best practices and tools for both FPC development and the transition to a guided-client choice model of distribution in food pantries.

**Trial registration:**

ClinicalTrials.gov; NCT03566095. Retrospectively registered on June, 21, 2018.

## Background

Food security has been defined as “access by all people at all times to enough food for an active, healthy life” [1] and “includes the ready availability of nutritionally adequate, safe foods, and the assured ability to acquire them in socially acceptable ways.” [2] Rural communities (often characterized by a population of 2500 persons or less) [3] experience unique barriers to food access when compared to urban areas, including, but not limited to, access and affordability of fruits and vegetables, [4] lack of transportation, [5] and chronic disease [6]. Communities with increased food insecurity prevalence when compared with the national average, may have a higher need for nutrition assistance, specifically food pantry services [7]. Integration of guided client-choice within pantries, development of food policy councils (FPCs) in communities, and support from community coaches may be a way to address food insecurity in rural, Midwestern areas.

Most food pantry users are food insecure, which is linked with being overweight and is often associated with diet-related co-morbidities [8]. Food insecure individuals may avoid hunger by consuming low-cost and shelf-stable foods, eating a small variety of foods, and/or binging when food is abundant [9]. Because certain characteristics and food insecurity predispose individuals to diet-related co-morbidities, ensuring that food pantries are able to provide nutrient-rich foods to the food insecure population in rural communities is crucial. Guided client-choice is a model of distribution in which pantry clients choose the foods they would like from the pantry based upon family size and is formatted based upon USDA MyPlate [10]. The guided client-choice style of food distribution in food pantries has been shown to serve the needs of the food pantry clients better when compared to the traditional model of distribution (pre-selected box or bag) [11]. This model reduces the amount of waste spurred by unwanted food items not being used and offers a more dignified experience by allowing pantry clients to choose foods that will supplement their diets [11]. Thus, when using a guided client choice model of distribution, food pantries have the potential to address the nutritional needs of the food insecure population.

FPCs have the potential to improve the nutritional quality of available food, affect federal-, state-, and local-level policy, systems and environmental efforts, and connect a diverse network of ‘food’ stakeholders from the public, private and nonprofit sectors, which includes local food pantries [12, 13]. While FPCs have been shown anecdotally to be effective in addressing food system and food security issues in urban areas [12], effectiveness has not been scientifically quantified. Furthermore, the use of FPCs as an intervention to improve food security in rural communities has not been evaluated. Since FPCs are comprised of food systems stakeholders including food pantries, it is plausible that FPCs could support emergency food operations in rural communities, and positively impact food pantry client household food security.

Community coaching is a strategy that helps community leaders plan for and overcome challenges to community development [14]. Community development work is most effective when interventions are locally conceived, locally led, and consistent with the cultural identity of the community [15]. Community coaches can effectively support community development through capacity building, fostering a collaborative environment, problem solving, reframing operating systems, transitioning to new leadership, and negotiating partnerships. Previous coaching success in sustaining community change has led some Cooperative Extension systems to recognize community coaching as a viable approach to driving sustainable community work and to institutionalize coaching as a value-added component of their work. Thus, utilizing Extension Specialists/field staff as community coaches may be an effective way to aid in the development and formation of FPCs in rural communities as they seek to develop long-term visions and goals to improve food security.

Six states (Indiana, Michigan, Missouri, Nebraska, Ohio, and South Dakota) have combined efforts to implement an integrated, community-led intervention in diverse, rural, high poverty Midwestern counties to enhance food security, called *Voices for Food*. A trans-disciplinary team of specialists in nutrition, agriculture, youth, community development, evaluation, and researchers from the Cooperative Extension North Central Region, developed *Voices for Food*. *Voices for Food* is a five-year integrated Extension and research project guided by the Social-Ecological Model of Behavior Change^12^ that addresses the United States Department of Agriculture (USDA), Agriculture and Food Research Initiative (AFRI) Food Security Challenge Area. The project team uses a multifaceted approach to promote socio-ecological changes in rural communities to increase access to, availability of, and consumption of nutritious foods. The project team selected rural communities with high poverty rates to engage with community coaches in order to: 1) develop new or provide support to existing FPCs and 2) encourage FPCs to make socio-ecological changes in their communities to increase the availability of, and access to, healthy food, which includes transitioning food pantries toward guided client choice systems. The specific project hypotheses are that: 1) having Extension Educators/field staff engaged with communities as community coaches will lead to the establishment or strengthening of multi-stakeholder FPCs working on goals to improve healthy food access within the community, and 2) in those communities that have stronger FPCs that support food pantries in transitioning to a guided client choice model called *MyChoice*, there will be greater improvement in availability of healthy foods for pantry users leading to improvement in their food security and intake of healthy foods. The purpose of this manuscript is to describe the methodology used to achieve these goals and assess the identified hypotheses.

## Methods/design

### Study design

*Voices for Food* used a longitudinal matched intervention and comparison design. Two matched treatment and comparison communities per state (*n* = 24) were selected to participate in the study based upon community and food pantry attributes. Institutional Review Board approval was obtained for this study prior to all intervention activities.

**Fig. 1:**
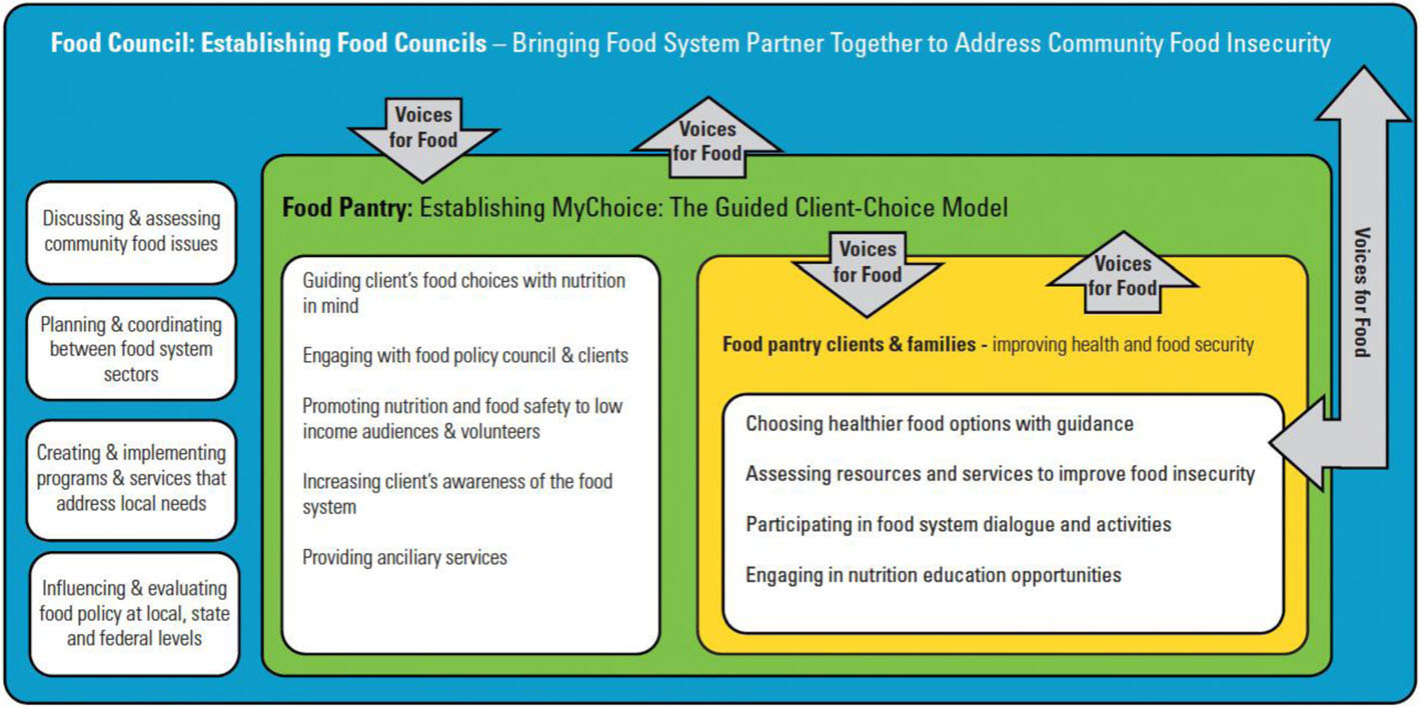
Voices for Food Model of Behavior Change

*Voices for Food* utilized the *Voices for Food Model of Behavior Change* (Fig. 1) to guide implementation activities. To address the hypotheses, *Voices for Food* focused on two key components that work together to impact food security in selected treatment communities: 1) Community coaching by Extension Educators/field staff, 2) provision of *Voices for Food* materials that describe, a) FPC development and/or support, and b) the transition to a *MyChoice* food pantry. In comparison communities, *Voices for Food* activities focused on one component: the provision of a *Voices for Food* materials that describes, a) FPC development and/or support, and b) development of a *MyChoice* food pantry*.* The notable difference between the treatment and comparison communities is that comparison communities did not receive coaching from a community coach throughout the implementation of Voices for Food. All participants in both treatment and comparison groups were blinded to the intervention. There were no circumstances in which unblinding was permissible.

### Materials

To guide the development or strengthening of FPCs in both intervention and comparison communities, the project team developed a the *Voices for Food: Food Council Creation Guide*, which includes information on the following topics: importance of engaging FPCs in food systems work; how to develop/sustain a FPC; networking and communicating with stakeholders; developing a FPC structure; partnering with food banks and pantries; working with the agricultural community; finances; and community food assessments. The guide also provides information regarding the opportunity to apply for mini-grant funds provided by *Voices for Food*. Mini-grant funds were available to all intervention and comparison communities, which required collaboration between the FPC and pantry and consistency with *Voices for Food* goals. All communities had the opportunity to submit a budget plan and justification, which was vetted and approved by the *Voices for Food* PD leadership team.

To guide the transition to *MyChoice* in the food pantry in both intervention and comparison communities, the project team developed a *Voices for Food: Food Pantry Toolkit* which includes information on the following topics: implementing the *MyChoice* guided client-choice model of distribution, a *Voices for Food* Ambassador’s Training (includes Nutrition Education [16], Food Safety Training [17], and Cultural Competency Training [18]), shelf talkers (labels), and USDA MyPlate materials. Nutrition education [16] were delivered in partnership with SNAP-Ed as an approved, evidence based curriculum was used for this portion [19, 20]. Both Food Safety [17] and Cultural Competency Training [18] were delivered and completed by pantry staff using the resources included in the toolkit.

### Identifying communities

Criteria were developed to identify eligible communities for the study. Eligible communities were those located in counties defined as non-metro with poverty rates > 16% [21], those that have a Cooperative Extension presence, do not have a well established FPC or similar organization in place, and do not already have a full guided client-choice model in their local food pantry in place.

### Matching intervention and comparison communities

Prior to recruitment, intervention and comparison communities were matched based upon the criteria for identifying communities and the characteristics of the local food pantry in the community. Pantry characteristics that aided in matching communities were: physical location, distance to another potential pantry, administering organization, hours and days of operation, level of client-choice, interest in study participation and working with Cooperative Extension, number of households served, an estimate of long-term pantry users (greater than 2 years), pounds of food distributed per month, government commodity program assistance (e.g. The Emergency Food Assistance Program [TEFAP], Food Distribution Program on Indian Reservations [FDPIR], and Commodity Supplemental Food Program [CSFP], food bank partnership, infrastructure and capacity (storage, shelving, etc.), and predominant racial/ethnic group served at the pantry.

## Community & pantry recruitment

### Food pantry

Pantry directors were invited to participate through an invitational letter. Upon confirmation, the coach scheduled an in-person meeting with the pantry director to discuss project details and explain the Memorandum of Understanding (MOU), which was signed upon agreement to participate. Once the treatment food pantries were recruited, project staff followed the same protocol in matched comparison communities.

### Community champion

Community champions were recruited to receive coaching for FPC development in the implementation of *Voices for Food* in both treatment and comparison communities. With the assistance of the pantry directors, a list of potential local community champions and stakeholders was created. A letter was sent to the potential community champion in each community to introduce the project, identify the participating pantry, to inquire about interest in assisting with *Voices for Food* and to request confirmation of participation. Upon confirmation of participation, an in-person meeting was scheduled by the coach with the community champion to discuss project details and the MOU, which was signed upon agreement to participate.

## Measures

### Organization/scheduling data collection

Quantitative and qualitative data were collected Fall Year 02 (Y02; pre-intervention), Fall Year 04 (Y04; mid-intervention) and Fall Year 05 (Y05; post-intervention) from community stakeholders, food pantry directors, food pantry staff/volunteers, and food pantry clients. A longer timeframe between pre-intervention and mid-intervention data collection was observed to allow more time for the treatment activities to be implemented.

### Determination of sample size

Assessments were conducted at three levels, which include community stakeholders, food pantry organization (food pantry, staff/volunteers), and food pantry clients. Convenience samples of community stakeholders, food pantry directors, and food pantry staff/volunteer participants were recruited and included all members of FPCs and all directors, staff/volunteers of the food pantries participating in the study. Sample size was based on the food pantry client expected improvement of 1 in adult food security score from pre-intervention to post-intervention on the food security scale (continuous score from 0 to 10). This unit of change in food security is practically meaningful as it could mean a change in food security level, for example an improvement from very low to low food secure which would indicate a change from not eating enough to an adequate amount of food. Pantry client participants were nested within food pantries. Previous research [22, 23] suggests SD (Y04-Y02) = SD (Y05-Y02) = 2.6 (using the largest conservative estimate) when Y02, Y04, Y05 represent the scores across years and SD represents the standard deviation. Thus, a power analysis was completed where n = the number of paired intervention (trt) and comparison (ctrl) pantries as: Var (estimated trt Y04- Y02 - estimated ctrl Y04-Y02) = 2 * 2.61*2.61/ *n* = 13.6242/n for a total of 224 clients, or 14 participants per pantry. An approximate 30% loss to follow up each year [19] was expected for a total estimate of 40 participants per pantry at baseline to be followed longitudinally throughout the project. An additional 40 new pantry clients were recruited at Y04 and Y05 in order to maintain power to detect a change in food security score in a cross-sectional study design.

### Participant recruitment

Community stakeholders, pantry directors, pantry staff/volunteers, and pantry clients were recruited to participate in this study. All participants were enrolled using the IRB-approved consent process. Community stakeholders were recruited for survey completion during the first FPC meeting after the intervention started, as they joined the council and at each data collection time point. If a FPC had not formed, community stakeholders were recruited from the community. Food pantry directors were recruited to complete surveys and were identified by the coach. Food pantry staff/volunteers were identified by pantry directors and were recruited for survey completion at each data collection time point. All food pantry staff/volunteers, directors and community stakeholders received $10 gift cards to local grocery stores when possible at each data collection time point for completing surveys.

Pantry client participants were recruited with fliers posted throughout the community, in high-traffic areas within the pantry, and by word-of-mouth. On the day of data collection, food pantry clients were screened for eligibility to ensure they were legal adults, could read and speak English, had visited the pantry more than once in the last 12 months, had access to a computer or telephone to complete follow up assessments, and desired to participate in the study. Food pantry clients were eligible to receive $30 total in stipends to a local grocery store at each data collection time point if all surveys were completed.

### Assessment tool descriptions

Eleven assessment tools were used to evaluate the effectiveness of the intervention as a whole. All questionnaires were pilot tested for feasibility prior to collecting pre-intervention data. Where possible, the project team used previously validated assessment tools or questions. All assessments were completed at pre-, mid- and post-intervention unless otherwise indicated. Table 1 summarizes the assessment tools and data collection schedule.

**Table 1:** Description of Assessment Tools

	Pre	Mid	Post	Subject
Community Level				
Community Stakeholders Survey	X	X	X	Project Staff
Food Council Implementation Tracking Form	X	X	X	Community Champion
Training Tracking Form	Ongoing			Pantry Director Community Champion
Food Pantry Organization Level				
Food Pantry Director Survey	X	X	X	Project Staff
Food Pantry Staff/Volunteer Survey	X	X	X	Project Staff
Food Pantry Inventory Log	X	X	X	Project Staff
Food Pantry MyChoice Observation Tool	X	X	X	Project Staff
Food Pantry Client Level				
Food Pantry Client Survey	X	X	X	Project Staff
ASA24® Dietary Recall	X	X	X	Project Staff
Participant Food Box Content Log	X	X	X	Project Staff

### Community-level assessment tools

#### Community stakeholders survey

A 23-question *Community Stakeholders Survey* assessed community stakeholder perceptions in participating communities. This survey collected demographic information, perceptions of food security in the community, past experience with FPCs or similar organizations and feedback on the *Voices for Food: Food Council Guide*, and experience with the community coach.

#### Food council implementation tracking form

A three-part *Food Council Implementation Tracking Form* tracked changes occurring in the FPC including: activities and accomplishments. Additionally, FPCs will provide key documents developed during the intervention, including meeting agendas, meeting minutes, press releases, organizational charts, mission/vision statements and strategic plans to the research team.

#### Training tracking form

A 5-question *Training Tracking Form* tracked the number of trainings completed from the *Voices for Food: Food Pantry Toolkit*, training topics, numbers of attendees, curriculum used, who was in attendance (e.g. FPC members, food pantry clients, pantry staff, etc.), and the use of resources from the *Voices for Food: Food Pantry Toolkit*. Throughout the intervention, project staff documented the nature of Extension coaching assistance provided to the intervention food pantries.

### Food pantry organization level assessment tools

#### Food pantry director survey

The 42-question *Food Pantry Director Survey* collected key information about the food pantry director and the food pantry including: demographic information, perceptions on community food security, and information about the food pantry.

#### Food pantry staff/volunteer survey

A 34-question *Food Pantry Staff/Volunteer Survey* collected key information about food pantry staff/volunteers and the food pantry including: demographic information, community perceptions on food security, perceptions of their abilities to interact with clients, and information about the food pantry.

#### Food pantry inventory log

One *Food Pantry Inventory Log* was maintained in a Microsoft Access database per state for all pantries to document the type and amounts of foods in stock at each food pantry site. Food pantry inventory data was collected on a date when the director indicated inventory will be relatively high (e.g. soon after food comes in from the food bank). The logs will be assigned United States Department of Agriculture [USDA] food codes in Food and Nutrient Database for Dietary Studies 5.0 that can be used to determine healthfulness of available foods [24].

#### Food pantry MyChoice observation tool

A 15-question *Food Pantry MyChoice Observation Tool*, was completed by project staff, documented the extent to which key components of the *MyChoice* food pantry model were physically in place at the pantry and part of the food, food display, and distribution process.

### Food pantry client-level assessment tools

#### Food pantry client survey

A 54-question *Food Pantry Client Survey* collected information from pantry clients including: demographic information, household information, and participation in food assistant programs such as Supplemental Nutrition Assistance Program [SNAP], household food security, where food is purchased, experience at the food pantry, perception of pantry food selection, and perception of food-related community activities. The United States Household Food Security Survey Module [25, 26] is embedded into the *Food Pantry Client Survey* and will assess food security in pantry clients. Individuals will be classified as very low food secure, low food secure, marginal food secure and high food secure.

#### ASA 24-h dietary recall

Dietary intake data (24-h recalls) were collected using the Automated Self-Administered 24-h (ASA24®) Dietary Assessment Tool, version 2014 and 2016, developed by the National Cancer Institute, Bethesda, MD [27]. The ASA24® was completed three times within the same week, on two week days and one weekend day. The first ASA24® was scheduled to be completed in-person with project staff on the day of the pantry visit. The second and third ASA24® recalls were self-completed or completed with project staff by telephone interview. The ASA24® allows for calculation of the Healthy Eating Index score, which is a measure of diet quality [28].

#### Participant food box content log

One *Pantry Food Box/Food Bag Log* was maintained in Microsoft Access per state for all pantries, which detailed all food items the pantry clients receive during that pantry visit. Participant Food Box Content Logs were collected on the day of data collection in the pantry. The logs will be assigned USDA food codes that can be used to determine healthfulness of the foods that clients chose or were given at the pantry [24].

### Statistical analysis

The first hypothesis is that having Extension Educators/field staff engaged with communities as community coaches will lead to the establishment or strengthening of multi-stakeholder FPCs working on goals to improve healthy food access within the community. This hypothesis will be assessed using several tools including the *Food Council Implementation Tracking Form*, *Community Stakeholders Survey,* Training *Tracking Form*, *Voices for Food Coaching Journal*, *Voices for Food Annual Budget Plan and Justification* and *Budget Follow-up Form*.

The second hypothesis is that in those communities that have stronger FPCs that support food pantries in transitioning to *MyChoice*, there will be greater improvement in availability of healthy foods for pantry users leading to improvement in their food security and intake of healthy foods. This hypothesis will be assessed using several tools including the *Food Pantry Inventory Log*, *Participant Food Box Content Log,* ASA24® [27] and *Food Pantry MyChoice Observation Tool.*

Descriptive statistics will be used to describe the study population at baseline. Analyses include examining changes in pantry food access/availability pantry client food security and dietary intake as a result of intervention or comparison group assignment. These outcomes will be compared longitudinally. T-tests and chi-square will be used for initial examination of mean and proportion differences in characteristics and outcome variables among treatment groups, followed by mixed-model regression analyses, which will allow adjustment for covariates and the examination of independent variables on outcomes.

### Study status

This study was conducted in rural, high poverty communities from 2014 to 2017. The study is currently ongoing with the final data collection concluding in November 2017, and analysis of hypotheses and main outcomes concluding in 2018.

## Discussion

There were four notable strengths of this study. First, the use of a multi-state collaborative team and the systems approach through the involvement of individuals with varying expertise and areas of interest contributed to a well-rounded, accurate protocol. Second, the use of a community-based approach that allowed the project team to meet each community where they were at in terms of readiness, and to recognize the individual strengths and weaknesses of each individual community and pantry strengthened this study. Each community was unique in where they were beginning and what they needed to progress. Allowing coaches to meet communities where they were at ensured that solutions were community based and relevant. Third, the use of evidence-informed and some evidence-based tools when possible and pre-testing the tools for feasibility, allowed for a comprehensive set of assessments to measure the effectiveness of the intervention as whole. Last, alignment with policy, systems, and environmental focuses for long-term applicability and sustainability in communities. The project team maintained a commitment to long-term sustainability and in turn promoted that through careful selection of community coaches and Extension presence in each community.

This study faced four main challenges. First, community-based work is time-consuming because of the effort needed for development of community relationships and partnerships, which impacted timelines for this project and posed additional challenges of keeping community stakeholders engaged over the long term. Timelines were extended and frequent communication with communities and partners was completed. Additionally, due the time consuming nature of this work, a longer timeframe between baseline and mid-point data collection was observed to allow time for treatment conditions to be implemented. Second, selecting sites that fit the criteria across six unique states and were similar enough for comparison was challenging but it was critical for study design and evaluation. Third, completion of a longitudinal study with a transient population was a challenge as the project team attempted to retain food pantry clients as study participants. The project team developed a protocol for maintaining updated contact information for participants, which included sending a flyer to all participants quarterly to request updated contact information. Last, the complexity of managing a large multi-level research study across six states was a challenge as we attempted to maintain fidelity to the protocol in all states with many personnel. To promote fidelity to the protocol, frequent, clear communication, trainings, video recordings and management strategies were used to provide consistency across the states involved in this study. Furthermore, during each data collection time-point, fidelity checks were completed with each state to assess fidelity to the protocol and mitigate any deviation from the protocol.

As a result of this study, Best Practices for utilizing health professionals in conjunction with the *Voices for Food: Food Council Guide* and the *Voices for Food: Food Pantry Toolkit* will be released for use by health professionals to 1) develop or strengthen FPCs in rural, high poverty communities, and 2) transition local food pantries to a *MyChoice* model of distribution in order to enhance food security. Furthermore, this study can be used as a framework for future policy, systems and environment work completed by health professionals throughout the United States.

## Abbreviations

FPC: Food Policy Council
